# Hepatoma-Targeted Radionuclide Immune Albumin Nanospheres: ^131^I-antiAFPMcAb-GCV-BSA-NPs

**DOI:** 10.1155/2016/9142198

**Published:** 2016-02-15

**Authors:** Mei Lin, Junxing Huang, Dongsheng Zhang, Xingmao Jiang, Jia Zhang, Hong Yu, Yanhong Xiao, Yujuan Shi, Ting Guo

**Affiliations:** ^1^Clinical Medical Institute, Taizhou People's Hospital Affiliated to Nantong University, Taizhou, Jiangsu 225300, China; ^2^Medical School, Southeast University, Nanjing, Jiangsu 210009, China; ^3^Jiangsu Key Laboratory for Biomaterials and Devices, Nanjing, Jiangsu 210009, China; ^4^Key Laboratory of Advanced Catalytic Material and Technology, Changzhou University, Changzhou, Jiangsu 213000, China

## Abstract

An effective strategy has been developed for synthesis of radionuclide immune albumin nanospheres (^131^I-antiAFPMcAb-GCV-BSA-NPs).* In vitro* as well as* in vivo* targeting of ^131^I-antiAFPMcAb-GCV-BSA-NPs to AFP-positive hepatoma was examined. In cultured HepG2 cells, the uptake and retention rates of ^131^I-antiAFPMcAb-GCV-BSA-NPs were remarkably higher than those of ^131^I alone. As well, the uptake rate and retention ratios of ^131^I-antiAFPMcAb-GCV-BSA-NPs in AFP-positive HepG2 cells were also significantly higher than those in AFP-negative HEK293 cells. Compared to ^131^I alone, ^131^I-antiAFPMcAb-GCV-BSA-NPs were much more easily taken in and retained by hepatoma tissue, with a much higher T/NT. Due to good drug-loading, high encapsulation ratio, and highly selective affinity for AFP-positive tumors, the ^131^I-antiAFPMcAb-GCV-BSA-NPs are promising for further effective radiation-gene therapy of hepatoma.

## 1. Introduction 

Undoubtedly, an ideal cancer treatment must meet two aspects: good therapeutic effect and no or little side effect [1, 2]. However, most current therapies, such as radiation and chemotherapy, destroy normal tissue and cause serious side effects while killing tumor cells. Traditional administration by intravenous injection ensures that drugs are uniformly distributed in the system. Generally, once administered, a drug undergoes many steps where loss can occur, including combination with plasma proteins, metabolism, and decomposition, before it gets to the tumor site. Only a small proportion of drugs finally reaches the tumor due to lack of specific affinity for tumor tissues or cells. This not only greatly decreases the therapeutic effect, but also increases the nonspecific side effect on normal tissue [3]. Traditional external radiation is currently well accepted as one of the most effective remedies for cancer, but it may inflict severe damage on normal tissue. Compared to external radiation, internal nuclide radiation provides prolonged low dose rate exposure and shows some advantages, but only a small minority of cancers can actively absorb nuclides unassisted. For example, thyroid carcinoma can take in ^131^I by itself, but most tumors are nonselective to this treatment. In recent years, suicide gene therapy has been explored for cancer treatment. Among suicide genes, herpes simplex virus type thymidine kinase (HSV-TK) is most commonly used. It can express thymidine kinase to convert nontoxic prodrug ganciclovir (GCV) into toxic GCV-TP to kill tumor cells by blocking DNA synthesis. Studies have shown that better curative effects can be obtained when the anticancer effects of the HSV-TK/GCV system are combined with radiotherapy [4]. In our previous study, we succeeded in constructing recombinant plasmids of pEgr1-HSV-TK and transferring them into hepatoma cells. Upon irradiation, radiation promoter Egr1 could induce HSV-TK gene to express efficiently and the encoded products could convert GCV into a tumor killing drug [4, 5]. However, the ultimate goal to kill the cancer without damage to normal tissue cannot be achieved unless the radiation is localized to the tumor site and suicide genes only express effectively in tumor cells not in normal cells or the prodrugs are delivered selectively to the tumor. Therefore, it is important to develop drugs or nuclides into tumor-targeted agents to improve curative effect and minimize side effect.

Monoclonal antibody (McAb) is a very powerful cancer-targeted tool and has been widely used in targeting treatment [6–9]. Owing to its high specificity, strong affinity for the corresponding tumor, and little injury to normal cells, great progress in cancer-targeted therapy has been made. Studies have shown that monoclonal antibodies can specifically target tumor cells with the corresponding antigen and can carry therapeutic agents such as nuclides or drugs to tumor site to kill the tumor [10, 11]. McAb has become a preferred choice for a guiding drug vector because of its unique superiority, but the lethality of drugs carried by a single monoclonal antibody molecule is poor.

Nanoparticle drug delivery system using nanospheres as delivery vectors can accommodate much more antitumor drug molecule and increase the drug-loading significantly. In particular, bovine serum albumin (BSA) nanospheres, which use BSA as vectors to encapsulate drugs, show very good qualities for a delivery vector including good stability, high drug-loading, and slow release. BSA nanospheres bearing paclitaxel, adriamycin, or nuclides (^125^I and ^188^R) showed a much improved antitumor effect [12–15]. As a result of good targeting, drug-loaded nanospheres cross-linked with monoclonal antibodies have a greater ability to kill target cells specifically [16, 17]. Drug-loaded immune nanospheres have been used to label or separate cells and diagnose or treat disease because of the antibody adsorbed on the particles which results in nanospheres' immunocompetence [18]. For example, if combined with fluorescent protein, drug-loaded immune nanospheres can be used for detection and diagnosis.

At present, nanosized BSA targeting agents mediated by McAb and radioimmune therapy are active areas in tumor-targeted therapy. Advances in protein cross-linking technology have paved the way for construction of radioactive targeting immune nanospheres by applying drug-loaded BSA nanospheres to radiation immunotherapy. As a type of specific tumor antigen in the membrane and cytoplasm of cells, *α*-fetoprotein (AFP) is positive in over 70% of primary hepatic carcinomas, but negative in normal liver or other normal tissues [19, 20]. Consequently, it is a good potential antigen for hepatic cancer treatment. Owing to its high specificity and affinity for AFP antigen in hepatic cancer cells, anti-AFP monoclonal antibody (antiAFPMcAb) can carry various “warheads” such as chemotherapy agents, radionuclides, or toxins to selectively attack AFP-positive cancer cells [21–23].

Based on the above, it can be conceived that if drug-loaded nanospheres are combined with ^131^I-labeled antiAFPMcAb, they could play a dual role of targeted radiotherapy and drug therapy on hepatoma. The drugs delivered in the nanospheres can be released slowly, which is crucial for improvement of the curative effect and reduction of side effect. If the drug is a prodrug such as GCV, in addition to the targeted radiation therapy, ^131^I can activate the promoter Egr1 to induce HSV-TK expression, converting GCV into a toxic drug for targeted radiation-gene therapy.

In the present study, ^131^I-antiAFPMcAb-GCV-BSA nanospheres targeted to AFP-positive tumors were constructed and their characteristics and hepatoma-targeting* in vitro* and* in vivo* were investigated, with the aim of providing theoretical and experimental insights for further high-efficiency radiation-gene therapy of hepatoma.

## 2. Material and Methods 

### 2.1. Main Material

DMEM, 0.25% trypsase/0.038% EDTA, and fetal bovine serum were purchased from Gibco; BSA, GCV, and SPDP (N-succinimidyl-3-(2-pyridyldithiol) propionate) were purchased from Sigma; ^131^I was purchased from Nanjing Senke Company; chloramine-T and glutaraldehyde were purchased from Jiaxing Chenlong Chemical Co. Ltd.; antiAFPMcAb was purchased from Shanghai Yemin Biotechnology Company; HepG2 cells (AFP-positive) and HEK293 cells (AFP-negative) were purchased from the Institute of Biochemistry and Cell Biology, Shanghai Institute of Biological Sciences, Chinese Academy of Sciences, China.

### 2.2. Preparation and Characterization of GCV-BSA-NPs

GCV-BSA-NPs were prepared by the desolvation method [24] as follows: (1) GCV solution with a concentration of 10 mg/mL was prepared; (2) 200 mg BSA was dissolved in 23 mL twice-distilled H_2_O; (3) 2 mL of (1) was added to (2) and the mixture was stirred magnetically. NaOH was used to adjust the pH of the solution to 9; (4) while being stirred magnetically, 100 mL EtOH was added slowly (1 mL/min) to (3), and the stirring was continued for additional 5 min; (5) 50 *μ*L of 25% glutaraldehyde was added to (4), and then the mixture was stirred magnetically for 24 h; (6) the solution was centrifuged, and the precipitate was washed three times with distilled water.

The morphology of GCV-BSA-NPs was checked by a transmission electron microscope (TEM) (JEM-200CX, Japan).

### 2.3. GCV Standard Curve

200 *μ*g/mL of GCV stock solution was used to prepare standard solutions with GCV concentrations of 10, 20, 30, 40, 50, and 60 *μ*g/mL. Using distilled water as a blank, the optical density (OD) values of each GCV standard solution were measured at a wavelength of 252 nm using a microplate reader (Multiskan MK3-353, USA). A standard curve was drawn, using the OD values as an ordinate and concentrations as abscissa, and a linear regression equation was established based on the data.

### 2.4. Drug-Loading (DL) and Encapsulation Ratio (ER) of GCV-BSA-NPs

10 mg GCV-BSA-NPs were transferred to a 50 mL volumetric flask with 0.5% pepsin solution, and the mixture was digested and dissolved in water bath at 37°C for 2 h. After being cooled to room temperature, distilled water was added to 50 mL, and then the solution was filtered, and the OD was measured at 252 nm, GCV content was calculated according to the above standard curve, and DL and ER were calculated according to the following formulas:

 Drug-loading efficiency = (GCV mass of the nanospheres/the total mass of the nanospheres) × 100%.
 Encapsulation ratio = (GCV mass of the nanospheres/the total mass of GCV) × 100%.

### 2.5. Preparation and Purification ^131^I-antiAFPMcAb

^131^I-antiAFPMcAb was prepared by chloramine-T method [25, 26] and purified on a gel chromatographic column. Trichloroacetic acid was used to precipitate the protein to test the labeling rate, radioactive concentration, and radioactive activity. The radiochemical purity was assessed by testing the chromatography paper.

### 2.6. Preparation, Purification, and Characterization of ^131^I-antiAFPMcAb-GCV-BSA-NPs

^131^I-antiAFPMcAb-GCV-BSA-NPs were prepared by the following chemical cross-linking. (1) ^131^I-antiAFP-McAb was modified by SPDP: 1 mL of purified ^131^I-antiAFP-McAb was dialyzed for 12 h and then its volume was adjusted to 4 mL with PBS. While being mixed magnetically, 13.7 *μ*L of SPDP solution in EtOH (20 mmol/L) was added to the mixture. After reaction for 30 min at room temperature, the reactant was transferred into a dialysis bag and dialyzed with acetate buffer (0.01 mmol/L, pH 4.5) to remove excess SPDP. Then it was enriched with PEG6000 to 4 mL and some DTT was added to make its concentration reach 50 mmol/L. Finally, the ^131^I-antiAFPMcAb-SH was obtained after this reaction mixture was dialyzed using PBS as dialyzate to remove redundant DTT; (2) 1 mg of GCV-BSA-NPs was dispersed in 3.0 mL acetic acid salt solution (pH 4.5) and then added to 50 *μ*L SPDP solution in EtOH (20 mmol/L). After being stirred for 30 min at room temperature, the reactant was centrifuged for three times for 1 min at 12,000 rpm and then washed with Hank's solution to remove redundant SPDP. Then the activatory albumin nanospheres (GCV-BSA-PDP-NPs) were obtained; (3) after (2) and (1) were mixed immediately and reacted for 15 h at 4°C with slow stirring, the crude ^131^I-antiAFPMcAb-GCV-BSA-NPs were obtained.

After the ^131^I-antiAFPMcAb-GCV-BSA-NPs were separated and purified, their labeling rate was measured by the same protocol with ^131^I-antiAFPMcAb. Their radiochemical purity was tested by paper chromatography at 0, 1, 6, 12, and 24 h in air at room temperature and at 1, 6, 12, and 24 h in serum incubated at 37°C.

A transmission electron microscope (TEM, JEM-200CX, Japan) was used to observe the morphology of ^131^I-antiAFPMcAb-GCV-BSA-NPs. The size distribution and average size of the particles were examined by a laser particle size analyzer (Malvern HPPS5001, England).

### 2.7. Cellular Uptake Experiment* In Vitro*

HepG2 cells and HEK293 cells in the logarithmic phase were digested with 0.25% trypsin and diluted into single cells (5 × 10^5^ cells/mL) with the fresh complete culture medium and then seeded in 24-well plates (1 mL/well). The ^131^I-antiAFPMcAb-GCV-BSA-NPs and ^131^I absorbed by HepG2 cells were grouped as (1) and (2); groups (3) and (4) were labeled as ^131^I-antiAFPMcAb-GCV-BSA-NPs and ^131^I absorbed by HEK293 cells, respectively. Three replicates were done in every group. The above cells were incubated in air containing 5% CO_2_ at 37°C. When the cells were attached well, the original culture medium in each well was replaced with 1 mL of fresh complete culture medium. 20 *μ*Ci of ^131^I-antiAFPMcAb-GCV-BSA-NPs was added to each well of groups (1) and (3), and 20 *μ*Ci of ^131^I was added to each well of groups (2) and (4). The plates continued to be incubated and the cell uptake rate of every group was tested at 0.5 h, 1 h, 2 h, 3 h, and 4 h. The culture medium in every well was taken out and the cells were washed three times with PBS. The culture medium and PBS used for washing were admixed and the radioactive counts of the mixture (C1) were measured by a *γ* counter. After the adherent cells in each well were lysed by lysis buffer (0.5 M NaOH + 1% SDS) and removed, each well was washed with PBS three times. Then the lysate and the corresponding PBS used for wash were mixed together and the radioactive counts of the mixture (C2) were measured by a *γ* counter. Cellular uptake rate was calculated using the following formula: cellular uptake rate = [C2/(C1 + C2)] × 100%. Radioactive uptake curves of ^131^I-antiAFPMcAb-GCV-BSA-NPs and ^131^I absorbed in HepG2 cells and in HEK293 cells at different time were drawn. This experiment was repeated three times.

### 2.8. Cell Retention Experiment* In Vitro*

Experiment grouping and the first half procedure were the same as those for cellular uptake experiment. Number of cells in each well in the cell retention experiment was 5 × 10^5^ and three replicates were done in every group. ^131^I-antiAFPMcAb-GCV-BSA-NPs and ^131^I were added into the corresponding wells. After incubation for 2 h, the culture medium of every well was removed and each well was washed three times with PBS. Then the same volume of fresh culture medium was added into each well and the plates continued to be incubated. The culture medium and cells in each well were collected together at 0.5 h, 1 h, 2 h, 4 h, and 6 h, and the total radioactive counts of every group were determined. After centrifugation for 10 min at 1000 rpm, the supernatant was removed and radioactive counts in cells from each well were measured. Radioactive retention rate was calculated by the following formula: retention rate (%) = ((radioactive counts in cells)/(radioactive counts in cells + radioactive counts in culture medium)) × 100%. The radioactive retention curves for ^131^I-antiAFPMcAb-GCV-BSA-NPs and ^131^I retained in HepG2 cells and HEK293 cells at different times were drawn, using time as the abscissa and retention rate as the ordinate. This experiment was performed three times.

### 2.9. Nude Mice Model of Transplanted Hepatocarcinoma

Female 6-week-old BALB/c nude mice with body weight of 20–22 grams, purchased from the Institute of Biochemistry and Cell Biology, Shanghai Institute of Biological Sciences, Chinese Academy of Sciences, China, were used for the experiments. All experiments were approved by the Animal Care Committee of Jiangsu Province and were performed in accordance with the institutional guidelines. All the mice were maintained in the sterile barrier system of Medical School, Southeast University, China. Exponentially growing HepG2 cells (2 × 10^6^ cells) were injected subcutaneously around the right posterior limb rump of the nude mice. All the mice were maintained in a sterile barrier system. After tumor diameters reached about 0.5 cm, the nude mice were used for the following experiments.

### 2.10. Radioactive Distribution Assay* In Vivo*

Twenty-four nude mice with tumors were randomly divided into two groups of twelve mice each: (1) ^131^I-antiAFPMcAb-GCV-BSA-NPs group and (2) ^131^I group. ^131^I-antiAFPMcAb-GCV-BSA-NPs and ^131^I (7.4 MBq/mouse) were injected into the tail vein of mice of group 1 and group 2. At 1 h, 8 h, 24 h, and 48 h after the injection, three mice were picked out randomly from group 1, and their blood was obtained by eyeball extraction. The same was done in group 2. All the mice were killed and their livers, spleens, kidneys, hearts, lungs, stomachs, intestines, muscles, bones, brains, and tumors were taken out to weigh and measure their radioactivity. Meanwhile, the radiocounts of the standard source were tested. The radioactive intake per gram of every organ (% ID/g) was calculated according to the following formula: ID/g = (tissue radiocounting (cpm)/tissue weight (g)/standard source radiocounting (cpm)) × 100%. T/NT (radioactivity ratio of tumor versus nontumor) was calculated with the following formula: T/NT = ID/g of tumor tissue/ID/g of nontumor tissue.

### 2.11. Statistical Analysis

Experimental values are reported as mean ± SD. The data were analyzed with the SPSS 16.0 program. A *p* value of <0.05 was considered significant.

## 3. Results 

### 3.1. Characteristics of ^131^I-antiAFPMcAb-GCV-BSA-NPs

**Figure 1:**
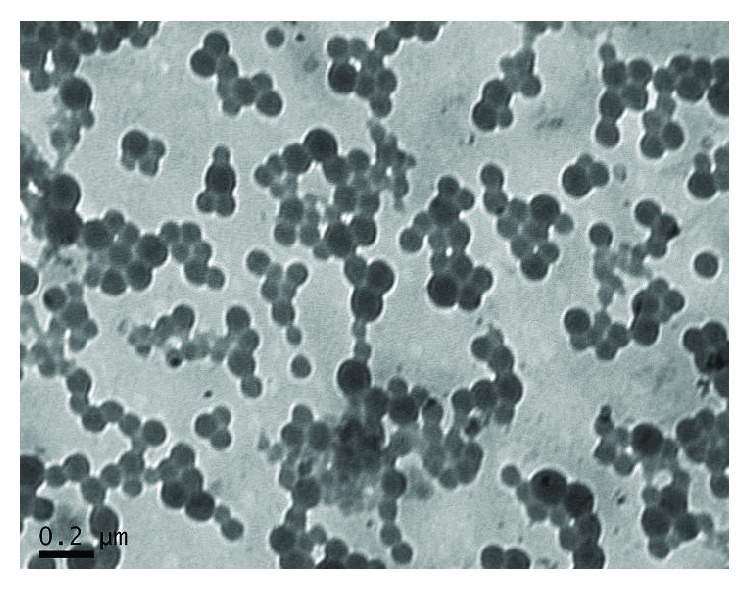
TEM image of GCV-BSA-NPs. GCV-BSA-NPs were nearly spherical, about 100–130 nm in diameter.

As shown in Figure 1, GCV-BSA-NPs were nearly spherical, about 100–130 nm in diameter and uniform in size. The effective drug-loading rate of GCV-BSA-NPs was 22.70%, and the GCV embedding rate was 70.63%.

As a rule, when radionuclide is employed in clinical treatment or* in vivo* analysis, its radiochemical purity is required to be more than 90% [27, 28]. In this study, chloramine-T was used to prepare ^131^I-antiAFPMcAb. The labeling yield was (72.97 ± 1.28)% and the radiochemical purity was (99.63 ± 0.11)%. ^131^I-antiAFPMcAb-GCV-BSA-NPs were obtained by connecting ^131^I-antiAFPMcAb to GCV-BSA-NPs by SPDP. The labeling yield of ^131^I-antiAFPMcAb-GCV-BSA-NPs was (61.5 ± 1.92)%. The radiochemical purity in air at room temperature for 0 h, 1 h, 6 h, 12 h, and 24 h was (96.05 ± 1.92)%, (94.87 ± 1.41)%, (93.60 ± 1.06)%, (91.71 ± 0.85)%, and (90.44 ± 0.28)%, respectively. The radiochemical purity in serum at 37°C for 1 h, 6 h, 12 h, and 24 h was (92.61 ± 1.63)%, (91.04 ± 0.92)%, (90.71 ± 0.56)%, and (90.11 ± 0.10)%, respectively. All were >90%, which met the requirement for the further radionuclide research* in vivo*.

**Figure 2:**
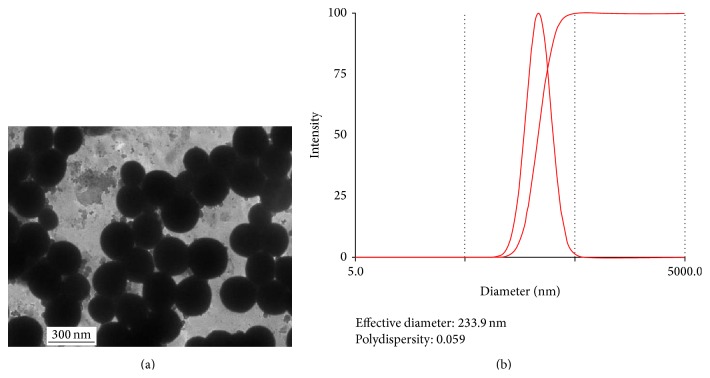
Size and distribution of ^131^I-antiAFPMcAb-GCV-BSA-NPs. (a) TEM image of ^131^I-antiAFPMcAb-GCV-BSA-NPs. The particles were approximately spherical and their diameters were about 220–280 nm. (b) Diameter and particle distribution of ^131^I-antiAFPMcAb-GCV-BSA-NPs examined by laser particle size analyzer. The average diameter was 233.9 nm. The polydispersity index (PDI) of the particles was 0.059.

The shape, the size, and diameter distribution of ^131^I-antiAFPMcAb-GCV-BSA-NPs were detected by TEM and laser particle size analyzer. As shown in Figure 2(a) (TEM image), ^131^I-antiAFPMcAb-GCV-BSA-NPs were approximately spherical and their diameters were about 220–280 nm. The average size of ^131^I-antiAFPMcAb-GCV-BSA-NPs examined by laser particle size analyzer was 233.9 nm, and the polydispersity index (PDI) of the particles was 0.059, indicating being largely uniform in size (Figure 2(b)).

### 3.2. Cellular Uptake and Retention* In Vitro*

To detect the targeting of ^131^I-antiAFPMcAb-GCV-BSA-NPs to hepatoma, we compared the uptake and the retention of ^131^I-antiAFPMcAb-GCV-BSA-NPs in AFP-positive HepG2 cells with those in AFP-negative HEK293 cells* in vitro*. As controls, the uptake and the retention of ^131^I in AFP-positive HepG2 cells and in AFP-negative HEK293 cells were also tested* in vitro*.

**Figure 3:**
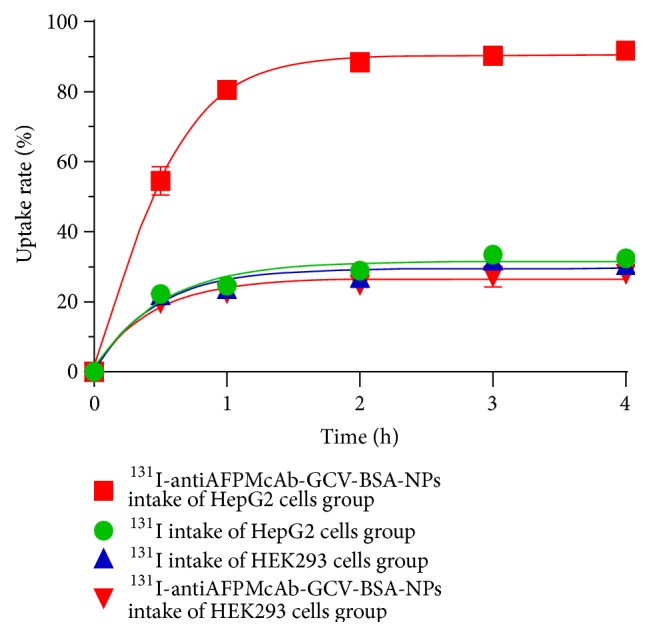
Uptake curves of ^131^I and ^131^I-antiAFP-GCV-BSA-NPs in HepG2 cells and HEK293 cells. The intake ratios of ^131^I-antiAFPMcAb-GCV-BSA-NPs in HepG2 cells group are far higher than those of ^131^I in HepG2 cells group, ^131^I-antiAFPMcAb-GCV-BSA-NPs in HEK293 cells group, and ^131^I in HEK293 cells group.

The cellular uptake test showed that the intake of ^131^I-antiAFPMcAb-GCV-BSA-NPs in HepG2 cells gradually increased, reaching (91.7 ± 1.9)% at 4 h, while the intake of ^131^I at 4 h was only (32.4 ± 2.1)%, far less than that of ^131^I-antiAFPMcAb-GCV-BSA-NPs at the corresponding time point. The intakes of ^131^I-antiAFPMcAb-GCV-BSA-NPs and ^131^I in HEK293 cells were both much less than that of ^131^I-antiAFPMcAb-GCV-BSA-NPs in HepG2 cells and there was little change with time. The statistics of ^131^I-antiAFP-GCV-BSA-NPs in HepG2 cells group differed significantly from those of the other three groups (*p* < 0.001 or 0.000) (Figure 3).

**Figure 4:**
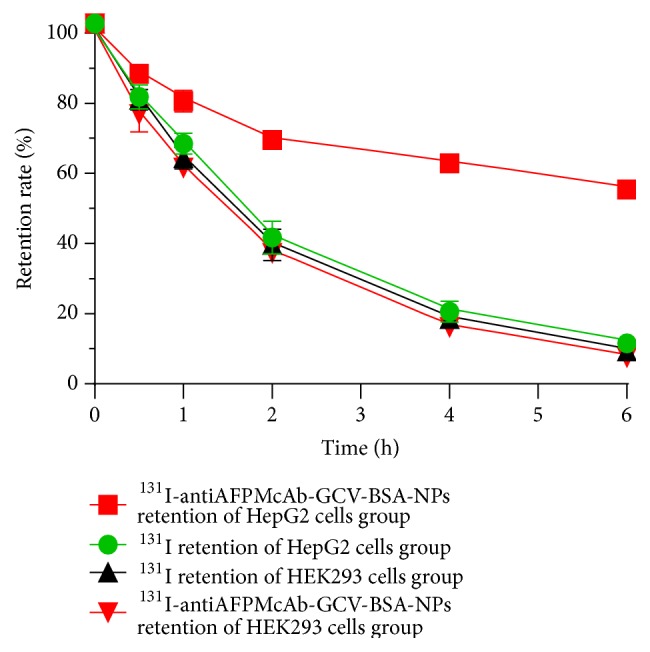
Retention curves of ^131^I and ^131^I-antiAFP-GCV-BSA-NPs in HepG2 cells and HEK293 cells. The radioactive retention rates of ^131^I-antiAFPMcAb-GCV-BSA-NPs in HepG2 cells group are obviously higher than those of other three groups at the same time point.

As shown in Figure 4, the radioactive retention rates of four groups decreased as time progressed, but the retention rate of ^131^I-antiAFPMcAb-GCV-BSA-NPs in HepG2 cells group was obviously higher than that of the other three groups (^131^I in HepG2 cells group, ^131^I-antiAFPMcAb-GCV-BSA-NPs in HEK293 cells group, and ^131^I in HEK293 cells group) at the same time point (*p* < 0.05, 0.01, 0.001, or 0.000), indicating that ^131^I-antiAFPMcAb-GCV-BSA-NPs are more easy to be taken in and retained in HepG2 cells with overexpressing AFP.

### 3.3. Radioactivity Distribution in Nude Mice with Transplanted Hepatoma

To validate the targeting of ^131^I-antiAFPMcAb-GCV-BSA-NPs to hepatoma* in vivo*, we investigated the nanospheres' dynamic radioactivity distribution in nude mice with transplanted hepatocellular carcinoma and calculated the radioactivity ratios of tumor versus nontumor (T/NT), using ^131^I alone as control. The results showed that the majority of ^131^I-antiAFPMcAb-GCV-BSA-NPs were distributed in blood, liver, and kidney at an early stage. As time progressed, the radioactivity in tumor tissue gradually increased while that in nontumor tissue decreased. ^131^I alone mainly concentrated in blood, liver, and kidney at early stages and distributed slightly in the tumor as time advanced, but its distribution in tumor tissue was far below that of ^131^I-antiAFPMcAb-GCV-BSA-NPs in tumor tissue at the same stage.  Table 1 shows T/NT values at different stages after ^131^I or ^131^I-antiAFPMcAb-GCV-BSA-NPs were injected into nude mice with transplanted hepatoma. All T/NT values in the groups of ^131^I-antiAFPMcAb-GCV-BSA-NPs gradually increased, indicating that ^131^I-antiAFPMcAb-GCV-BSA-NPs gradually gathered in tumor tissue. Compared to the ^131^I-antiAFPMcAb-GCV-BSA-NPs groups, all T/NT values in the ^131^I groups after 12 h were much lower at the same time point (*p* < 0.01, 0.001, or 0.000).

## 4. Discussion 

There are many methods with which to prepare albumin drug-loading nanospheres, such as ultrasonic emulsifying, desolvation, polymer dispersion, and mechanical grinding. Among these, the desolvation method is the simplest and rapidest. In the present study, we prepared GCV-BSA-NPs by this method. TEM revealed that the nanospheres were 100–130 nm in diameter, smaller than 200 nm of BSA-NPs prepared by ultrasonic emulsification, a technique developed by Müller et al. [29]. The small size as well as hydrophilic surface is helpful to decrease opsonization, which in turn assists particles not easily engulfed by macrophages.

Drug-loading (DL) is one of important indicators with which to evaluate carrier performance for loading drugs. Generally, the higher the DL, the less the carriers that have to be used, and the better the effect [30]. The encapsulation ratio (ER) refers to the percentage of the total mass of drug embedded in the carriers accounting for the total mass of drugs, which is an assessment of drug utilization, reflecting the maturity of the preparation technology. As a rule, the higher the ER, the less the free drugs, and the higher the utilization. In this study, the effective drug-loading rate of GCV-BSA-NPs was 22.70%, and the GCV embedding rate reached 70.63%.

^131^I has become one of the most commonly used radionuclides as a result of its superior properties, such as abundant sources, low price, and moderate half-life. In order to enhance targeting in radionuclide therapy, some special anti-McAb or ligand is often attached to the radionuclide. In the present study, chloramine-T technique was used to prepare ^131^I-antiAFPMcAb. The labeling yield was (72.97 ± 1.28)%, and the radiochemical purity reached (99.63 ± 0.11)%.

Protein connecting technology can link the drug-loading protein nanosphere and radiation immune antibody. The antibody can guide radionuclide-bearing drug-loading nanosphere to kill lesions selectively, and the nanosphere can thus play a double “bio-missile” role of radionuclide and drug. This dual local treatment can greatly improve killing effect for tumor and minimize systemic side effect.

SPDP is a different bifunctional protein cross-linking agent, at whose termini a disulfide pyridine group and amber imide resin group are sensitive to sulfhydryl and amino groups and can easily cross-link two proteins containing thiol and amino groups. We used SPDP in the current study to connect GCV-BSA-NPs and ^131^I-antiAFPMcAb, obtaining ^131^I-antiAFPMcAb-GCV-BSA-NPs. Their diameters were 220–280 nm with average diameter 233.9 nm. After separation and purification, the labeling yield of ^131^I-antiAFPMcAb-GCV-BSA-NPs was (61.5 ± 1.92)%. Their radiochemical purity both in air at room temperature and in serum at 37°C within 24 h was all in excess of 90%, indicating serum causes little damage to the radioactive stability. These ^131^I-antiAFPMcAb-GCV-BSA-NPs therefore can be used for targeting analysis and treatment* in vivo*.

Cellular uptake ratio is an important parameter with which to evaluate drug targeting* in vitro*. Generally, the more the uptake, the stronger the targeting. The uptake rate of ^131^I-antiAFPMcAb-GCV-BSA-NPs in HepG2 cells gradually increased with exposure, much greater than that of ^131^I alone in HepG2 cells at the corresponding time point. The uptake rate of ^131^I-antiAFPMcAb-GCV-BSA-NPs in HepG2 cells was far greater than that in HEK293 cells at the same time point. These indicate that ^131^I-antiAFPMcAb-GCV-BSA-NPs have good selectivity to HepG2 cells. It may result from the fact that the antiAFPMcAb on the nanospheres specifically binds to AFP antigen on the HepG2 cell membrane, which facilitates the cells to endocytose ^131^I-antiAFPMcAb-GCV-BSA-NPs, thus greatly improving the intake. The reason for the small change of ^131^I intake may be that its radioactivity in cells represents nonspecific adsorption.

After it is taken up into cells, some ^131^I-antiAFPMcAb-GCV-BSA-NPs may be retained in the cells; the rest is excreted. The retention time and the retention ratio are important factors influencing targeting. The cell retention test showed that the retention rate of ^131^I-antiAFPMcAb-GCV-BSA-NPs in HepG2 cells was much higher than that in HEK293 cells and clearly higher than that of ^131^I alone in HepG2 cells and HEK293 cells, suggesting better retention of ^131^I-antiAFPMcAb-GCV-BSA-NPs in HepG2 cells. This may also be related to antiAFPMcAb. To be specific, the antiAFPMcAb on the nanospheres specifically binds to AFP antigen in the cytoplasm of HepG2 cells. This leads to more ^131^I-antiAFPMcAb-GCV-BSA-NPs retained in HepG2 cells and longer retention time. By contrast, the entrance of ^131^I alone into HepG2 or HEK293 cells and ^131^I-antiAFPMcAb-GCV-BSA-NPs into HEK293 cells is passive, and they may quickly flush out of the cells.

The nuclides or drugs carried by nanospheres play a crucial role in killing tumor cells, and their concentration and residence time in tumor cells and T/NT are all crucial for the curative effect. T/NT is one of the main parameters which embody the ability of an antibody to concentrate specifically in tumor tissue. It reveals the relative ratio of affinities for antibody to tumor tissue and antibody to nontumor tissue. The nuclide distribution experiments* in vivo* showed that while most of ^131^I-antiAFPMcAb-GCV-BSA-NPs and ^131^I alone were distributed in blood, liver, and kidney at an early stage, the radioactivity in tumor tissue gradually increased and that in nontumor tissue decreased as time progressed in the ^131^I-antiAFPMcAb-GCV-BSA-NPs group. In comparison, radioactive distribution had no clear difference between tumor tissue and nontumor tissue in ^131^I alone group; both presented a gradually decreasing trend as time passed. Consistently, T/NT values of each ^131^I-antiAFPMcAb-GCV-BSA-NPs group all showed an obviously increasing trend as time went on, significantly higher than that of the corresponding ^131^I group at 12, 24, and 48 h. These data demonstrate that ^131^I-antiAFPMcAb-GCV-BSA-NPs have a strong selectivity to AFP-positive hepatoma and the high affinity between antiAFPMcAb and AFP antigen can assist ^131^I and GCV carried by the nanospheres to concentrate in hepatoma tissue.

In summary, ^131^I-antiAFPMcAb-GCV-BSA-NPs have been prepared successfully for the first time in the present study.* In vitro* and* in vivo* experiments confirmed the highly selective targeting of ^131^I-antiAFPMcAb-GCV-BSA-NPs to AFP-positive hepatocellular carcinoma tissue or cells. This is significant for the further radiation-gene therapy to selectively treat hepatoma and also provides a new strategy for other cancer-targeted treatment.

## Figures and Tables

**Table 1:** T/NT of  ^131^I-antiAFPMcAb-GCV-BSA-NPs and  ^131^I in nude mice with transplanted hepatoma (mean ± SD, *n* = 3).

Time	4 h		12 h		24 h		48 h	
Groups	^131^I-antiAFP-GCV-BSA-NPs	^131^I	^131^I-antiAFP-GCV-BSA-NPs	^131^I	^131^I-antiAFP-GCV-BSA-NPs	^131^I	^131^I-antiAFP-GCV-BSA-NPs	^131^I
Heart	1.26 ± 0.04^1^	1.12 ± 0.14	2.26 ± 0.17^2^	1.16 ± 0.07	4.16 ± 0.21^3^	1.30 ± 0.21	8.77 ± 0.19^4^	1.65 ± 0.26
Liver	0.46 ± 0.04^5^	0.35 ± 0.04	0.86 ± 0.06^6^	0.39 ± 0.09	1.36 ± 0.16^7^	0.41 ± 0.02	2.35 ± 0.40^8^	0.46 ± 0.06
Spleen	1.02 ± 0.06^9^	0.91 ± 0.05	1.78 ± 0.06^10^	0.97 ± 0.12	3.08 ± 0.16^11^	1.14 ± 0.12	9.02 ± 1.42^12^	1.57 ± 0.12
Lung	1.19 ± 0.04^13^	1.07 ± 0.14	2.26 ± 0.20^14^	1.18 ± 0.24	4.65 ± 0.52^15^	1.29 ± 0.10	9.38 ± 2.21^16^	1.90 ± 0.60
Kidney	0.64 ± 0.03	0.56 ± 0.10	1.29 ± 0.07^17^	0.66 ± 0.17	2.31 ± 0.08^18^	0.77 ± 0.10	5.91 ± 0.29^19^	0.99 ± 0.21
Stomach	1.22 ± 0.11^20^	0.98 ± 0.11	2.37 ± 0.03^21^	1.18 ± 0.06	5.11 ± 0.45^22^	1.53 ± 0.28	9.75 ± 1.80^23^	1.99 ± 0.24
Intestine	1.58 ± 0.08^24^	1.25 ± 0.17	3.04 ± 0.03^25^	1.40 ± 0.18	7.44 ± 1.01^26^	2.07 ± 0.16	12.29 ± 0.56^27^	2.45 ± 0.45
Brain	3.85 ± 0.19^28^	2.58 ± 0.41	8.76 ± 0.20^29^	3.28 ± 0.82	15.67 ± 2.10^30^	3.42 ± 0.36	30.68 ± 5.36^31^	3.32 ± 0.41
Bone	0.97 ± 0.05	0.89 ± 0.12	2.24 ± 0.15^32^	1.00 ± 0.29	4.22 ± 0.15^33^	1.24 ± 0.06	7.05 ± 0.42^34^	1.52 ± 0.42
Muscle	1.16 ± 0.04	1.08 ± 0.04	2.74 ± 0.21^35^	1.34 ± 0.20	5.65 ± 0.57^36^	1.74 ± 0.21	10.88 ± 0.53^37^	2.09 ± 0.36
Blood	0.18 ± 0.01	0.15 ± 0.01	0.40 ± 0.03^38^	0.19 ± 0.03	0.66 ± 0.03^39^	0.22 ± 0.04	1.70 ± 0.12^40^	0.33 ± 0.08

^1^ *p* < 0.05 versus the heart group of 4 h ^131^I; ^2^ *p* < 0.01 versus the heart group of 12 h ^131^I; ^3^ *p* < 0.001 versus the heart group of 24 h ^131^I; ^4^ *p* < 0.000 versus the heart group of 48 h ^131^I; ^5^ *p* < 0.05 versus the liver group of 4 h ^131^I; ^6^ *p* < 0.01 versus the liver group of 12 h ^131^I; ^7^ *p* < 0.001 versus the liver group of 24 h ^131^I; ^8^ *p* < 0.000 versus the liver group of 48 h ^131^I; ^9^ *p* < 0.05 versus the spleen group of 4 h ^131^I; ^10^ *p* < 0.001 versus the spleen group of 12 h ^131^I; ^11^ *p* < 0.000 versus the spleen group of 24 h ^131^I; ^12^ *p* < 0.000 versus the spleen group of 48 h ^131^I; ^13^ *p* < 0.05 versus the lung group of 4 h ^131^I; ^14^ *p* < 0.001 versus the lung group of 12 h ^131^I; ^15^ *p* < 0.000 versus the lung group of 24 h ^131^I; ^16^ *p* < 0.000 versus the lung group of 48 h ^131^I; ^17^ *p* < 0.001 versus the kidney group of 12 h ^131^I; ^18^ *p* < 0.000 versus the kidney group of 24 h ^131^I; ^19^ *p* < 0.000 versus the kidney group of 48 h ^131^I; ^20^ *p* < 0.05 versus the stomach group of 4 h ^131^I; ^21^ *p* < 0.001 versus the stomach group of 12 h ^131^I; ^22^ *p* < 0.000 versus the stomach group of 24 h ^131^I; ^23^ *p* < 0.000 versus the stomach group of 48 h ^131^I; ^24^ *p* < 0.05 versus the intestine group of 4 h ^131^I; ^25^ *p* < 0.000 versus the intestine group of 12 h ^131^I; ^26^ *p* < 0.000 versus the intestine group of 24 h ^131^I; ^27^ *p* < 0.000 versus the intestine group of 48 h ^131^I; ^28^ *p* < 0.01 versus the brain group of 4 h ^131^I; ^29^ *p* < 0.000 versus the brain group of 12 h ^131^I; ^30^ *p* < 0.000 versus the brain group of 24 h ^131^I; ^31^ *p* < 0.000 versus the brain group of 48 h ^131^I; ^32^ *p* < 0.001 versus the bone group of 12 h ^131^I; ^33^ *p* < 0.000 versus the bone group of 24 h ^131^I; ^34^ *p* < 0.000 versus the bone group of 48 h ^131^I; ^35^ *p* < 0.001 versus the muscle group of 12 h ^131^I; ^36^ *p* < 0.000 versus the muscle group of 24 h ^131^I; ^37^ *p* < 0.000 versus the muscle group of 48 h ^131^I; ^38^ *p* < 0.001 versus the blood group of 12 h ^131^I; ^39^ *p* < 0.000 versus the blood group of 24 h ^131^I; ^40^ *p* < 0.000 versus the blood group of 48 h ^131^I.
